# Soil suction dataset from a lime & cement treated embankment, from 2010 to 2023

**DOI:** 10.1016/j.dib.2025.111506

**Published:** 2025-03-24

**Authors:** Yasmina Boussafir, Dimitri Mercadier, Christophe Piquet

**Affiliations:** a Gustave Eiffel University, GERS, Soils Rocks & Geotechnical structures Laboratory, 5 Bd Newton, F77454 Marne-la, Vallée cx2, France; b Cerema Rouen, DERDI, CER, 10 chemin de la poudrière, CS 90245, F-76121 Le Grand-Quevilly Cedex, France

**Keywords:** Suction, meteorology, Lime treated soil, Cement treated soil, Silt, Clay, Daily monitoring

## Abstract

An experimental embankment was built in 2010 in the frame of TerDOUEST Project [1]. Treated silt and clay from the site near Héricourt in France, were used in four sections approximately 50 meters length, and 5 meters high. The first meter at the base of the embankment is buried in the soil, below the ground, to study soil-water table interaction. In this site, a water table is located at approximately 1 to 2 meters depth and is registered with a piezometric probe. The last four meters of Héricourt embankment is above the ground level. The slope of this earth structure is V1:H2. One side of the embankment, has been built with the Low Plastic silt classified A2 according to the French standard NF P11-300 [2], treated for one third with 2% of quick lime, and another third section with 3% of cement (previously CEMII). The last third section was not treated. The other side of the embankment used clay, considered as a Plastic Clay and classified A4 [2]; one third has been treated with 4% of quick lime, another third section, with 2% of quick lime and 3% of cement, and the last third was not treated. In each of the treated sections, sensors were buried at 0.25 - 0.50 and 0.75 m depth in the slope, recording volumetric water content and suction for soil-atmosphere interactions studies. Other sensors recorded the volumetric water content only, in the core of the embankment, its base and the platform [3]. All data are available from 2010 to 2023. A weather station recorded precise meteorological data from 2010 to 2013.

The goal of this real size embankment was to evaluate the sustainability of treated soils used in earth structures and to test the re-use of very plastic clay thanks to adapted soil treatment. After more than 10 years old, this structure is stable, even if intrinsec characteristics may evolve [4].

Specifications Table

**Table:** 

Subject	Geotechnical Engineering and Engineering Geology.
Specific subject area	Evolution from 2010 to 2023, of physical features of compacted and treated Soils, in an in-situ embankment built in 2010 with clay and silt, treated with lime and/or cement, using suction probes, meteorological and piezometric devices – TerDOUEST project*.*
Type of data	Tables .xls and .csv format and Graphs .xls format..
Data collection	In the frame of Terdouest Project (ANR2007 - PGCU 006, from 2008 to 2013) almost 160 sensors buried in 2010 in treated soils, collected continuous data from 2010 to 2023, every day as well as Meteorological data in the same time. These data are useful to determine the sustainability of soil treatment over time in a continental climate (East of France) in the different part of an embankment, built according French recommendations. Graphs provided in this data collection are of great interest for assessing the effect of meteorology on soil response. Each couple of treatment/soil provide a different response to the same climatic environment. Sometimes monitoring missed energy or the collect did not work. That is why an average value of the four daily values of suction (one data every six hours) has been calculated using a python code. The mean suction value of each day was the best format for standardising departure data, the number of which could vary from one day to the next. They are in the excel and csv files provided with this document and plotted in graphs in the excel file only. When there is no data, for example during the first year of monitoring in 2010, the day is missing in the data sheet.
Data source location	Data are collected from an experimental embankment, located in Héricourt (Haute-Saône department, 70), France, near the National Road RN19. - City/Town/Region : Héricourt, Haute-Saône department, Bourgogne-Franche-Comté region - Country: France - Latitude and longitude (and GPS coordinates, if possible) for collected samples/data: 47°35′10.2 “N 6°44′26.5 “E
	
	
	
	

Data accessibility	Repository name: https://entrepot.recherche.data.gouv.fr/ Data identification number: https://doi.org/10.57745/AYRZUU Direct URL to data: … Instructions for accessing these data: …
Related research article	none*.*

## 1 Value of the Data

- **Why are these data useful?**

These data provide from a long term monitoring of suction along the slope of an embankment. Data were recorded simultaneously with volumetric water values (publication in progress) and meteorological data (2010-2013). Some values of settlement are available at the base of the embankment (publication in progress). Soil Temperatures are recorded in the same probe as the volumetric water content and sometime with temperature probes alone.

**Fig. 1:**
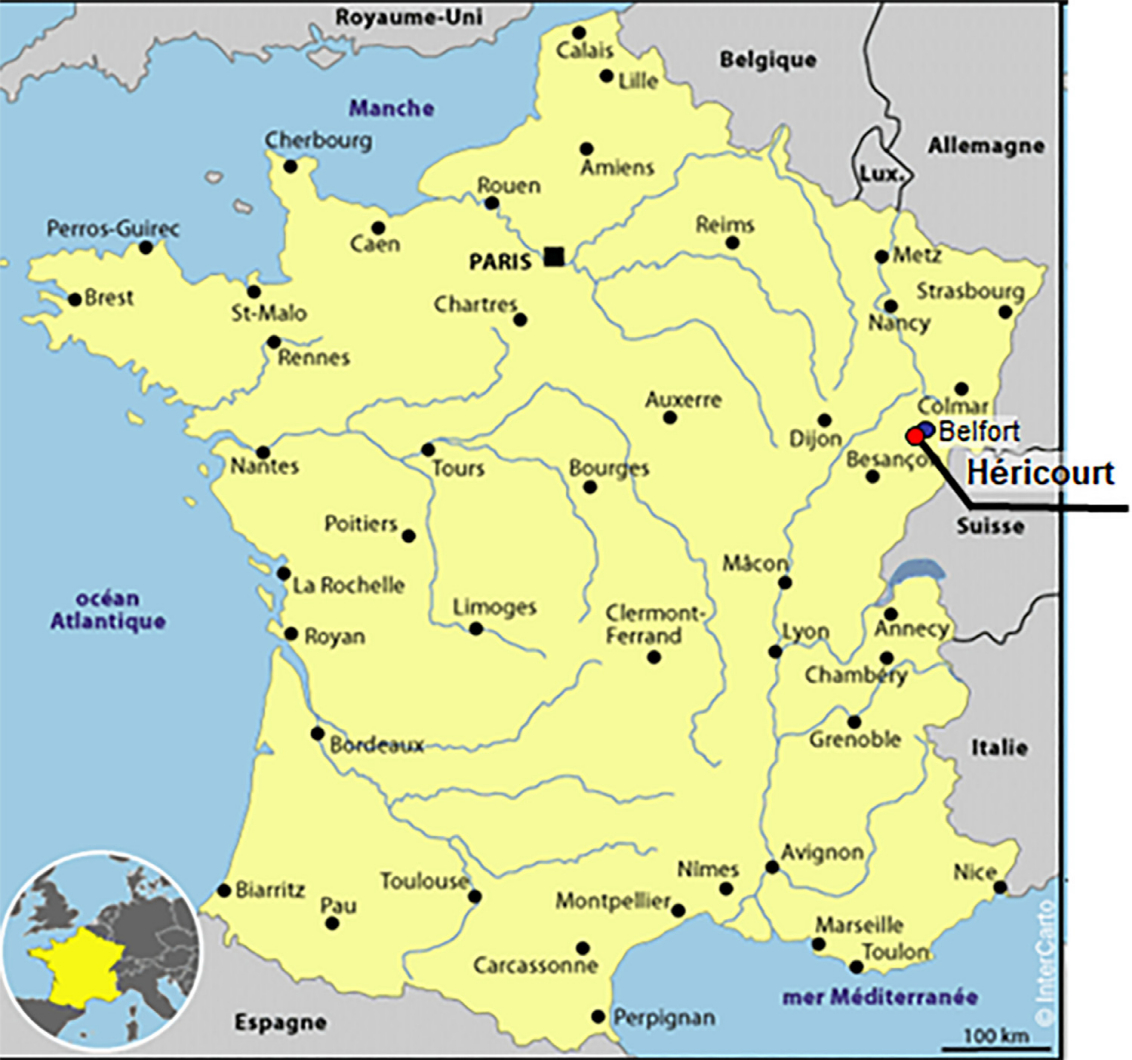
Location map of the instrumented site*.*

This data sheet is useful to evaluate the soil-atmosphere interaction during 13 years and explore the meteorological impacts on suction variations beyond the slope surface. They allow observation of seasonal effects of wetting and drying. This is useful for sustainability approaches, climate adaptation, and long-term performance evaluation for those who want to use treated silt and plastic clay in 4 meters high embankment. It is interesting to observe that response of each type of soil and kind of treatment is different due to different suction and unsaturated hydraulic conductivity.

The methodology of compaction is published in [[1], [2], [3], [4], [5],^1^]; performance of the compacted soil during earthworks and the initial values of dry density and gravimetric water content is presented in [6]. The performance of the embankment is discussed in [3,7,8,9]. The laboratory results previously to earthworks are presented in [5].

- **Who can benefit from these data?**

The data are available to any researchers/engineers who want to evaluate the performance of treated and compacted soils during ten years in a real size earth structure of five meters high, a water table near the topsoil, under a continental climate (East of France).

These information are also useful for stakeholders threatened with the technics of in situ soil-treatment. The stability of the earth structure is satisfying during the thirteen years of monitoring, even with a water table near ground level and a wet and cold climate.

- **How can these data be used/reused for further insights and/or development of experiments?**

Comparison with core sampling would greatly help to evaluate the sustainability of soil-treatment during 13 years, in the Hericourt climate which is a typical oceanic climate in France with wet and cold winters and short summers

## 2 Background

The original motivation was to proof the sustainability of soil treatments used for roads and railways embankment. The TerDOUEST project gathered 12 partners and aimed to answer this question. A specific motivation pointed out the ability to re-use high plastic clay in small embankments despite their high water sensitivity and swelling-shrinkage behaviour, thanks to adapted soil-treatments. The TerDOUEST partners worked from 2008 to 2013 on four topics. The third one (so-colled “part C”) had to build an experimental embankment, to register in situ information related to the long-term behaviour of the treated material in a real size earth structure. This data set collection is a result of the part C of Terdouest project. Many other data still exist to describe the behaviour the embankment.

## 3 Data Description

Files are available in excel (.xls) including data and graphs, and (.csv) for data only.

Sensors are burried in different layers. Each compacted layer is named depending on its vertical location in the embankment:

• 12 layers in the embankment named R0 to R11 (R="Remblai" in French, i.e. embankment layer), 0 = base and 11 = top of the embankment, approximately 0.30 m thick each;
• 3 layers in the upper part of the embankment named PST0 to PST2 (PST="Partie Supérieure des Terrassements" in French, i.e. upper part of embankment, approximately 1.00 meter thick)
• 1 layer for the capping layer named CDF0 (CDF="Couche De Forme" in French, i.e. capping layer)
• 1 layer for the foundation layer named ASS0 (ASS="Assise" in French, i.e. foundation layer)

Two piezometers are located at the toe of the embankment, one on the silty side and one on the clay side of the embankment. Two others piezometers are located at the crest of the embankment, on each side of the clay or silt side. The depth of the bottom of each piezometer is approximately 2 meters in the ground. They record the level of La Lizaine river water table.

**Fig. 2:**
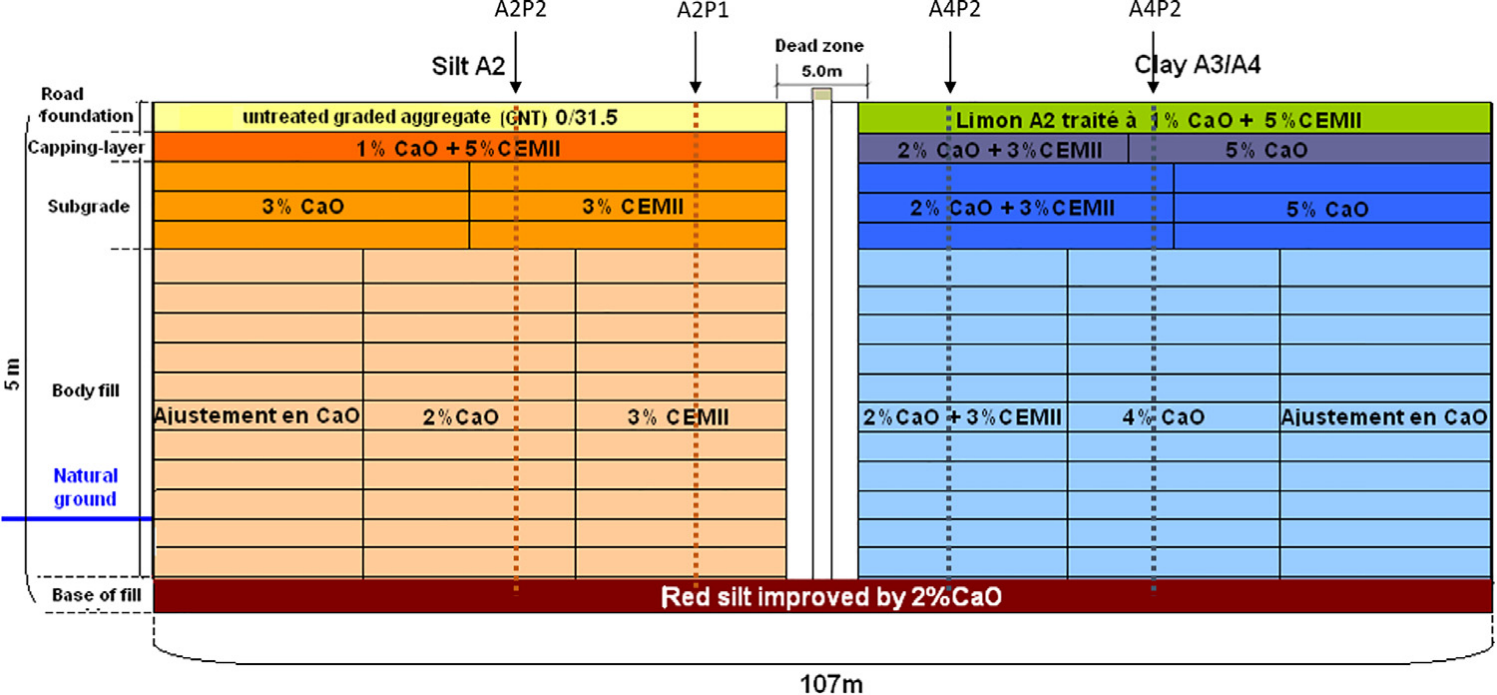
Front view of the embankement, location of the four instrumented profiles (green dashed line in clays and red in silts) in the different parts of the treated soils.

All the sensors were gathered on four vertical profiles (Fig. 2). The four profiles are named as follow (Fig. 1, Fig. 2):

- The French classification of the soil :
  - A4 for highly plastic clays,
  - A2 for silts
- The number of the profile :
  ○ 1 in the cement treated part of the embankment
  ○ 2 in the lime treated part of the embankment

For example, in the A4P2 profile, data recorded the sensors buried in the highly plastic clays treated with lime, and all the sensors in this vertical profile.

Fig. 9 provides a simple summary of the location of the sensors. A more detailed pdf file is in the data repository.

### 3.1 Summary of the data repository

- 8 excel files of daily Suction data over 13 years: June 2010 to August 2023 for the 4 treatment modalities (A4P1, A4P2, A2P1, A2P2):
  - 4 files to represent the values as a function of depth at -0.25, -0.50 and -0.75 m horizontally below the surface of the slope (that means not including the topsoil thickness), at the level of layer R8
  - 4 files to represent the values as a function of position on the slope and at the same depth (-0.25m below the surface of the slope) in layers R4, R5, R6 and R8.

In the 8 Excel files, graphs are provided. They represent evolution of suction in function of time, from June 2010 to August 2023, for each type of treatment (in cement treated soil: profiles P1 and in lime treated soil: profiles P2) and type of soil (clay-A4 and silt-A2):

  - graphical representation of suctions for each year from June 2010 to August 2023 for the 4 treatment modalities A4P1, A4P2, A2P1 and A2P2:
    - values as a function of depth on the R8 layer only, at -0.25, -0.50 and -0.75 m horizontally bellow the topsoil
    - values according to position on the slope, at -0,25 m horizontally below the topsoil in each layer, at the level of layers R4, R5, R6 and R8
- 4 csv files, gathering all the succion data for each A2P1, A2P2, A4P1 & A4P2 profile, that means the sensors in the slope and in the depth, with their location in each layer. In each file, temperature of a lonesome sensor of temperature is given, at -0,25 m depth in the layer R8.
- GPS coordinates of the site but not of the sensors (because the sensors were installed after backfilling): N47°35′09.89" - E6°44′26.35
- 1 excel file with weather data for the site: June 2010 to October 2013 (see also Météo-France data for Luxeuil -if free)
- 4 excel containing piezometric data from the four piezometers installed in the embankment, from June 2010 to October 2023
  In the 4 Piezometric excel files, from 2010 to 2023, two of which track water level variations for the treated silt modality (A2) and two of which track water level variations for the treated clay modality (A4), the graphs illustrate :
  - variations in the water level in the ground as a function of time, of one piezometers installed at the bottom (B) of the slope and the second one at the head (H) of the slope
- 2 csv file, gathering piezometric data for each A2 & A4 part of embankment, including those at the bottom and the head of the silt A2 or the clay A4.
- 1 pdf file with a diagram location of all sensors and their original ID, for each of the four profiles.

Other existing files: compaction per layer with LVDT data, topographic surveys of the various layers of compacted soil, topographic surveys of embankments + 3 monitored over time, accelerometer measurements under compactor load, temperatures, image, etc., all publication in progress.

## 4 Data from the Treated Silty-Soil (A2)

### 4.1 File « GsucA2P1_slope_mean_day.xls »

**Fig. 3:**
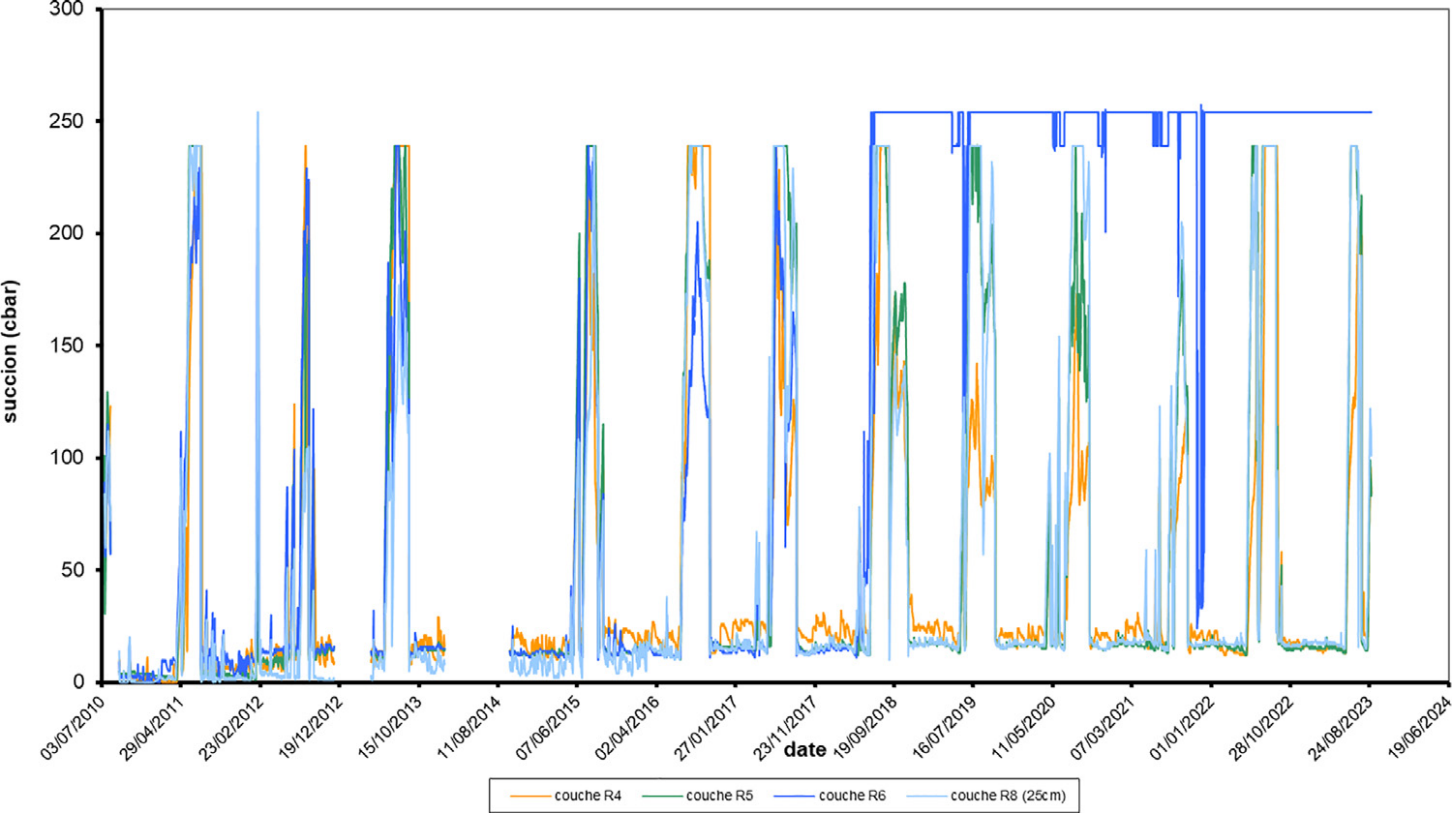
Example of suction data from 2010 to 2023, monitored in the slope of the cement treated silt part of the embankment (A2P1), at various heights from the ground level in layer R4 (∼0.60 m)- R5 (∼0.90 m)- R6 (∼1.20 m) & R8 (1.80 m) but the same depth (0.25 m).

The Excel file contains 29 sheets:

- 1 to 13: GsucA2P1_slope2010 to GsucA2P1_slope2023: 13 graphs for each year from 2010 to 2023, suction measured in the cement treated silt, on the slope
- 14: GsucA2P1_peau: one graph with all suctions from 2010 to 2023
- 15 to 28: Suction_2010 to Suction_2023: each sheet provides the individual data of daily suction for each year from 2010 to 2023.
- 29 Suction_all: this sheet provides all the data of daily suction from 2010 to 2023 (Fig. 3).

### 4.2 File « GsucA2P1_depth_mean_day.xls »

**Fig. 4:**
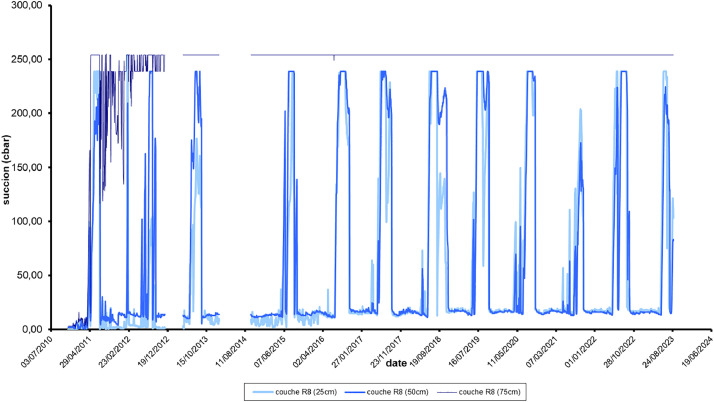
Example of suction data from 2010 to 2023, monitored in the depth of the cement treated silt part of the embankment (A2P1), in the layer R8, at 0.25 – 0.50 and 0.75 m horizontally. When the suction exceeds 200 kPa, the signal is capped.
- 33: Data_year: this sheet provides the option needed for the excel graph. It provides the x-axis value for a smart representation of one year from January 1st (minimum value) to December 31st (maximum value), and the increment (scale) for one month

this file contains 33 sheets:

- 1: recap : this sheet gives the name of each sensor and the corresponding location
- 2: place : this sheet illustrates all cross section of each profile and the sensors location
- 3 to 16: GsucA2P1_prof2010 to GsucA2P1_prof2023: 13 graphs for each year from 2010 to 2023, suctions measured in the depth of the layer R8
- 17 : GsucA2P1_slope: one graph with all suctions from 2010 to 2023
- 18 to 31: Suction_2010 to Suction_2023 : each sheet provides the individual data of daily suction for each year from 2010 to 2023.
- 32: Suction_all: this sheet provides all the data of daily suction from 2010 to 2023 (Fig. 4)

### 4.3 File « GsucA2P1_mean_day.csv »

The “GsucA2P1_ mean_day.csv” file contains only one sheet with the raw data of a temperature's probe at -0,25 m depth in the layer R8; and suction probes values for all sensors monitoring the A2P1 profile (that means the cement treated silt part of the embankment).

### 4.4 File « GsucA2P2_slope_mean_day.xls »

29 sheets:

- 1 to 13: GsucA2P2_slope2010 to GsucA2P2_slope2023: 13 graphs for each year from 2010 to 2023, suction measured in the lime treated silt, along the slope
- 14: GsucA2P2_slope: one graph with all suctions from 2010 to 2023
- 15 to 28: Suction_2010 to Suction_2023: each sheet provides the individual data of daily suction for each year from 2010 to 2023.
- 29 Suction_all: this sheet provides all the data of daily suction from 2010 to 2023

### 4.5 File « GsucA2P2_depth_mean_day.xls »

this file contains 33 sheets:

- 1: recap: this sheet gives the name of each sensor and the corresponding location
- 2: place: this sheet illustrates all cross section of each profile and the sensors location
- 3 to 16: GsucA2P2_prof2010 to GsucA2P2_prof2023: 13 graphs for each year from 2010 to 2023, suctions measured in the depth of the layer R8
- 17: GsucA2P2_slope: one graph with all suctions from 2010 to 2023
- 18 to 31: Suction_2010 to Suction_2023: each sheet provides the individual data of daily suction for each year from 2010 to 2023.
- 32: Suction_all : this sheet provides all the data of daily suction from 2010 to 2023
- 33: Data_year: this sheet provides the option needed for the excel graph. It provides the x-axis value for a smart representation of one year from January 1st (minimum value) to December 31st (maximum value), and the increment (scale) for one month

### 4.6 File « GsucA2P2_mean_day.csv »

The “GsucA2P2_mean_day.csv” f file contains only one sheet with the raw data of a temperature's probe at -0,25 m depth in the layer R8; and suction probes values for all sensors monitoring the A2P2 profile (that mean the lime treated silt part of the embankment).

## 5 Data from the Treated Clayey-Soil (A4)

### 5.1 File « GsucA4P1_slope_mean_day.xls »

29 sheets:

- 1 to 13: GsucA4P1_slope2010 to GsucA4P1_slope2023: 13 graphs for each year from 2010 to 2023, suction measured in the cement treated clay, along the slope
- 14: GsucA4P1_slope: one graph with all suctions from 2010 to 2023
- 15 to 28: Suction_2010 to Suction_2023: each sheet provides the individual data of daily suction for each year from 2010 to 2023.
- 29 Suction_all: this sheet provides all the data of daily suction from 2010 to 2023

### 5.2 File « GsucA4P1_depth_mean_day.xls »

this file contains 33 sheets:

- 1: recap : this sheet gives the name of each sensor and the corresponding location
- 2: place: this sheet illustrates all cross section of each profile and the sensors location
- 3 to 16: GsucA4P1_prof2010 to GsucA4P1_prof2023: 13 graphs for each year from 2010 to 2023, suctions measured in the depth of the layer R8
- 17: GsucA4P1_slope: one graph with all suctions from 2010 to 2023
- 18 to 31: Suction_2010 to Suction_2023: each sheet provides the individual data of daily suction for each year from 2010 to 2023.
- 32: Suction_all : this sheet provides all the data of daily suction from 2010 to 2023
- 33: Data_year: this sheet provides the option needed for the excel graph. It provides the x-axis value for a smart representation of one year from January 1st (minimum value) to December 31st (maximum value), and the increment (scale) for one month

### 5.3 File « GsucA4P1_mean_day.csv »

The “GsucA4P1_ mean_day.csv” file contains only one sheet with the raw data of a temperature's probe at -0,25 m depth in the layer R8; and suction probes values for all sensors monitoring the A4P1 profile (that mean the cement treated clay part of the embankment).

### 5.4 File « GsucA4P2_slope_mean_day.xls »

this file contains 29 sheets:

- 1 to 13: GsucA4P2_slope2010 to GsucA4P2_slope2023: 13 graphs for each year from 2010 to 2023, suction measured in the lime treated clay, on the slope
- 14 : GsucA4P2_slope: one graph with all suctions from 2010 to 2023
- 15 to 28: Suction_2010 to Suction_2023: each sheet provides the individual data of daily suction for each year from 2010 to 2023.
- 29 Suction_all: this sheet provides all the data of daily suction from 2010 to 2023

### 5.5 File « GsucA4P2_depth_mean_day.xls »

this file contains 33 sheets:

- 1: recap: this sheet gives the name of each sensor and the corresponding location
- 2: place: this sheet illustrates all cross section of each profile and the sensors location
- 3 to 16: GsucA4P2_prof2010 to GsucA4P2_prof2023: 13 graphs for each year from 2010 to 2023, suctions measured in the depth of the layer R8
- 17: GsucA4P2_slope: one graph with all suctions from 2010 to 2023
- 18 to 31: Suction_2010 to Suction_2023: each sheet provides the individual data of daily suction for each year from 2010 to 2023.
- 32: Suction_all: this sheet provides all the data of daily suction from 2010 to 2023
- 33: Data_year: this sheet provides the option needed for the excel graph. It provides the x-axis value for a smart representation of one year from January 1st (minimum value) to December 31st (maximum value), and the increment (scale) for one month

### 5.6 File « GsucA4P2_mean_day.csv »

The “GsucA4P2_mean_day.csv” file contains only one sheet with the raw data of a temperature's probe at -0,25 m depth in the layer R8; and suction probes values for all sensors monitoring the A4P2 profile (that mean the lime treated clay part of the embankment).

## 6 Meteorological Data

### 6.1 File “Data_meteos_site_2010_2013.tab”

This contains four sheets:

- 1 to 4: terDouest_METEO2010 to terDouest_METEO2013

Each sheet provides data of:

- rainfall (PLUVIO, column B) in mm
- wind speed (ANEMO, column C) in m/s
- soil temperature (TEMPSOL, column D) in Celsius degree (°C)
- wind direction (GIROUETT, column E) in degree / north
- light energy (LIGHT, column F) in kW/m2
- air temperature at 1,0 m height from soil surface (TAIRHAUT, column G) in°C
- Relative humidity at 1,0 m height from soil surface (RH.HAUT, column H) in %
- Relative humidity at 0,5 m height from soil surface (RH.BAS, column I) in %
- air temperature at 0,5 m height from soil surface (TAIR.BAS, column J) in°C
- air pressure (BAROMETR, column K) in hPa

for each year from 2010 to 2013. The day and time of acquisition appear in column A.

Data were acquired every half hour. For other data, the nearest official meteorological stations are LUXEUIL and MONTBELIARD.

## 7 Piezometric Data

### 7.1 File « Piezo_1_A4H_moyenne_jours.xls »

The excel file in French contains seventeen (17) sheets:

- 1 to 14 sheets : 14 graphs entitled “Graph_piezo_day_2010” to “2023”
- 1 graph for the 13 years “Graph_piezo_day_ALL”
- 1 sheet “piezo” with interpreted data of piezometric depth values in m NGF, at the head of the embankment built with Clay (A4H)
- 1 sheet “Raw_data” with raw data in Column B, and the coefficient values a, b and the level at the origin y0 which transform raw data into piezometry. The coefficient a transforms the voltage into water column (height in meters); b is the initial depth of the piezometer; y0 is the elevation of the piezometer head.

### 7.2 File « Piezo_2_A2H_moyenne_jours.xls »

The excel file in French contains seventeen (17) sheets:

- 1 to 14 sheets : 14 graphs entitled “Graph_piezo_data_2010” to “2023”
- 1 graph for the 13 years “Graph_piezo_day_ALL”
- 1 sheet “piezo” with interpreted data of piezometric depth values in m NGF, at the head of the embankment built with Silts (A2H)
- 1 sheet “Raw_data” with raw data in Column B, and the coefficient values a, b and the level at the origin y0 which transform raw data into piezometry. The coefficient a transforms the voltage into water column (height in meters); b is the initial depth of the piezometer; y0 is the elevation of the piezometer head.

### 7.3 File « Piezo_3_A4B_mean_day.xls »

The excel file contains seventeen (17) sheets:

- 1 to 14 sheets: 14 graphs entitled “Graph_piezo_day_2010” to “2023
- 1 graph for the 13 years “Graph_piezo_day_ALL”
- 1 sheet “piezo” with interpreted data of piezometric depth values in m NGF, at the bottom of the embankment built with Clay (A4B)
- 1 sheet “Raw_data” with raw data in Column B, and the coefficient values a, b and the level at the origin y0 which transform raw data into piezometry. The coefficient a transforms the voltage into water column (height in meters); b is the initial depth of the piezometer; y0 is the elevation of the piezometer head.

### 7.4 File “Piezo_4_A2B_moyenne_jours.xls”

The excel file in French contains seventeen (17) sheets:

- 1 to 14 sheets : 14 graphs entitled “Graph_piezo_day_2010” to “2023”
- 1 graph for the 13 years “Graph_piezo_day_ALL”
- 1 sheet “piezo” with interpreted data of piezometric depth values in m NGF, at the bottom of the embankment built with Silts (A2B)
- 1 sheet “Raw_data” with raw data in Column B, and the coefficient values a, b and the level at the origin y0 which transform raw data into piezometry. The coefficient a transforms the voltage into water column (height in meters); b is the initial depth of the piezometer; y0 is the elevation of the piezometer head.

### 7.5 File “Piezo_1_A4H_A4B_mean_day.csv »

The “Piezo_1.csv” file contains only one sheet with the interpreted data of piezometric depth values at the Bottom and the Head of the clay part of the embankment (A4). All depths are expressed in NGF elevation, the French reference.

### 7.6 File “Piezo_2_A2H_A2B_mean_day.csv »

The “Piezo_2.csv” file contains only one sheet with the interpreted data of piezometric depth values at the Bottom and the Head of the silty part of the embankment (A2). All depths are expressed in NGF elevation, the French reference.

### 7.7 Instrumentation diagram

This pdf file contains all the diagrams of the construction plans of the embankment: probes locations (with their names), layers location (with their names), in longitudinal and cross sections for all profiles: A4P1, A4P2, A4P3, A2P1, A2P2, A2P3; and one top view.

## 8 Experimental Design, Materials and Methods

All sensor cables are grouped together in a concrete nozzle located in the dead zone separating the two parts (silt and clay) of the embankment. At each layer, where sensors are buried, holes are drilled in the nozzle to bring all the cables up to the surface. On the surface of the backfill, a concrete slab is poured after the last layer has been laid, and a metal cabinet is fixed to the slab. The cables coming from the concrete nozzle enter the cabinet at its base through sheaths laid before the concrete is poured. The whole installation is protected by a fence.

The cables from the various sensors are grouped together and routed to a terminal block in the cabinet, to which the inputs of a Campbell CR3000 datalogger are connected. The whole system is powered by 24 VDC using two 12 V/80 Ah batteries. Eight time-stamped daily measurements (one every 3 hours) are taken for all the sensors and recorded in a file.

In order to save battery power, a sensor power supply wake-up system has been installed (relay controlled by an output from the control unit), which enables all the sensors to be powered only when measurements are being taken. The power supply is woken up 30 seconds before the measurements are taken and recorded. Transmission equipment (GSM modem) has been added to the system to enable on-demand communication with the central measuring station to retrieve data remotely without having to visit the site.

**Fig. 5:**
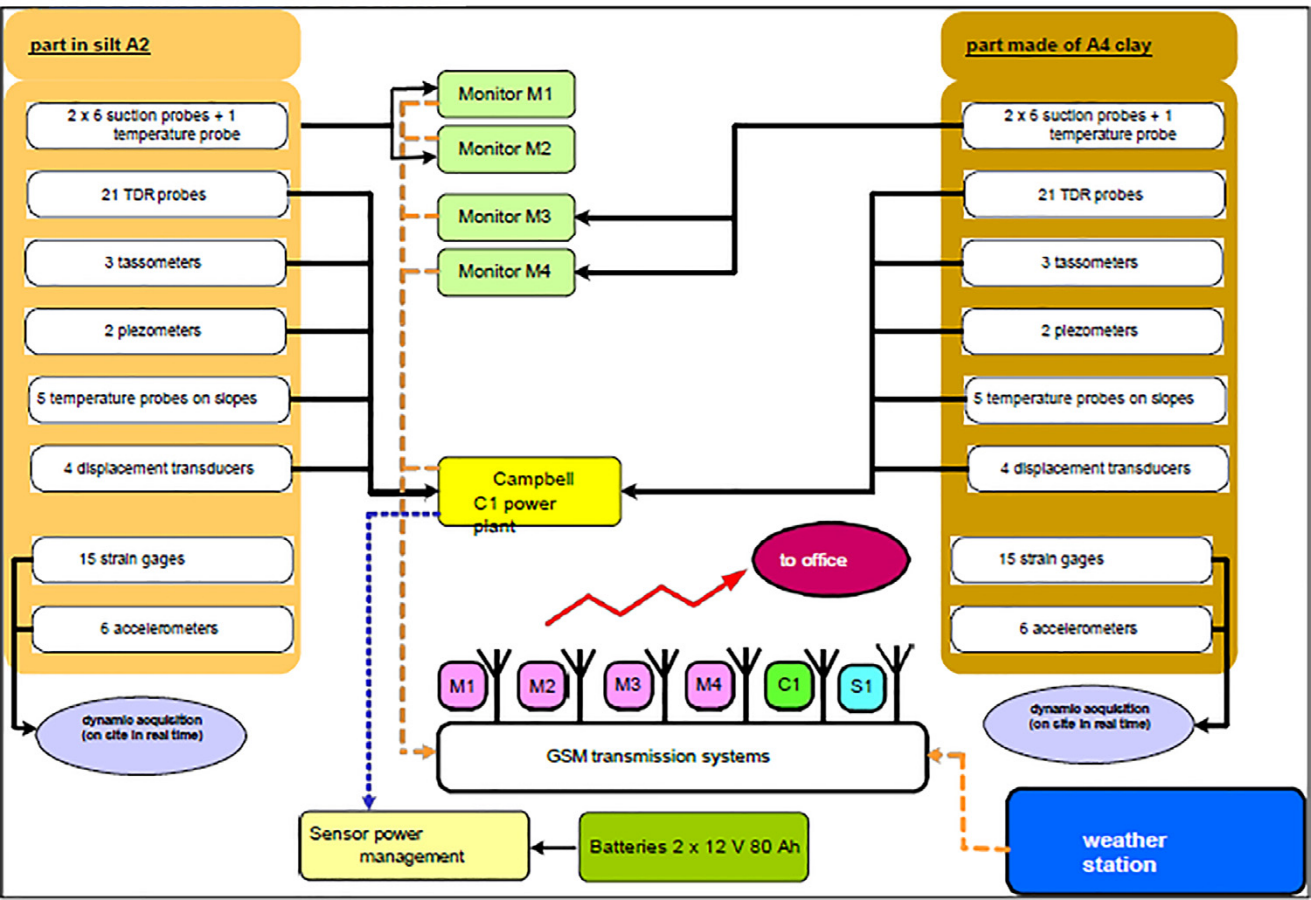
Data centralisation system overview.

The system for measuring suction on the embankments is independent of the power station. Each of the 4 batches of 6 suction probes is connected to an independent control unit which controls and records the data at a rate of approximately one measurement per day. A GSM modem is fitted to each of the 4 control units and sends the results to the recipient by e-mail. A synoptic of the data centralisation system is shown in Fig. 5.

**Fig. 6:**
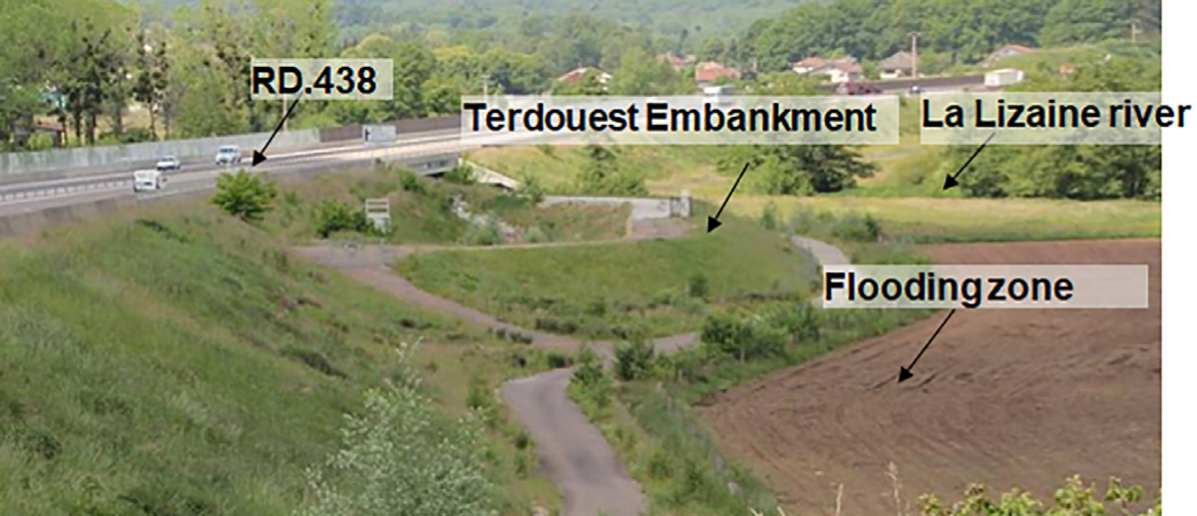
View of the embankment after earthworks (2010).

General overview of the site after Earthworks, in 2010 (Fig. 6). We can clearly see the National Road (RD.438), the shape of the drainage basin of the Road, and the Terdouest experimental embankment, the river La Lizaine on the back of the picture and its flooding area.

**Fig. 7:**
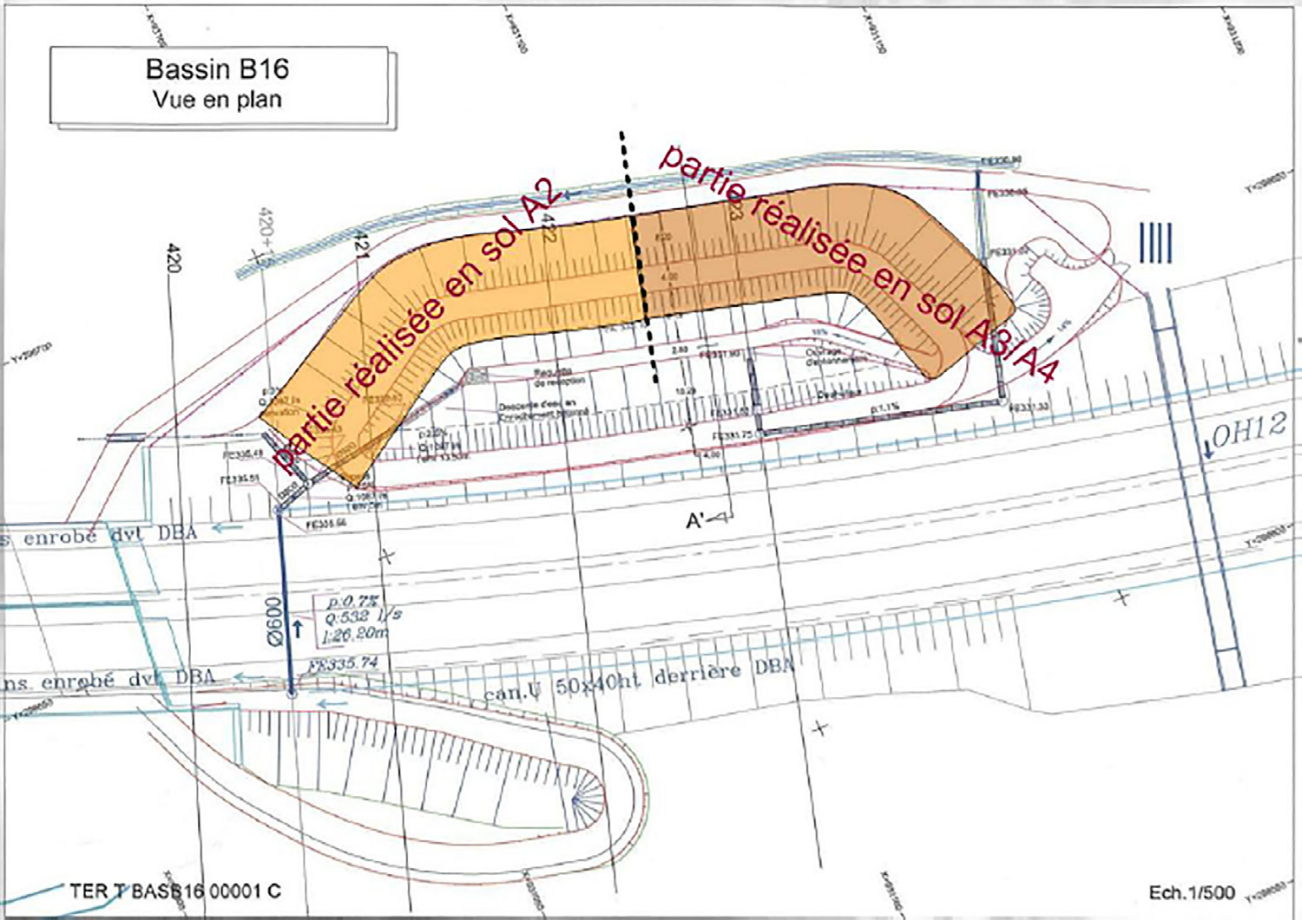
Layout of the two sections built with the two types of soil.

**Fig. 8:**
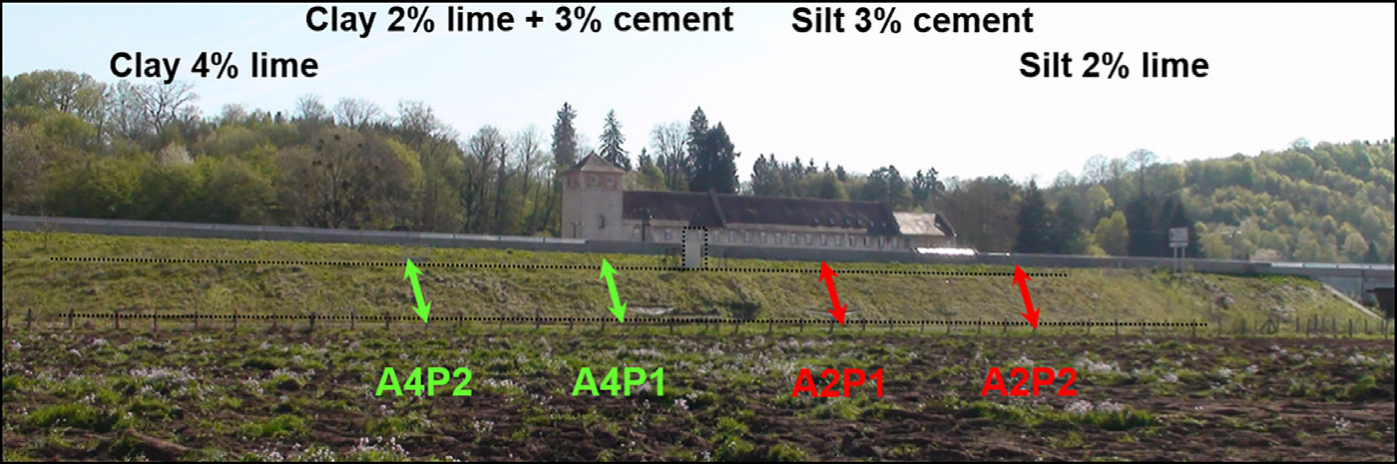
Front view of the embankment, location of the four instrumented profiles (in 2010).

Above (Fig. 7), a view from the top of the embankment : on the left side of the figure, the Silty soil (A2) and on the right side, the Clayey soil (A4). Bellow (Fig. 8) photo from the front of the embankment, in 2010, with the location of the four profile.

**Fig. 9:**
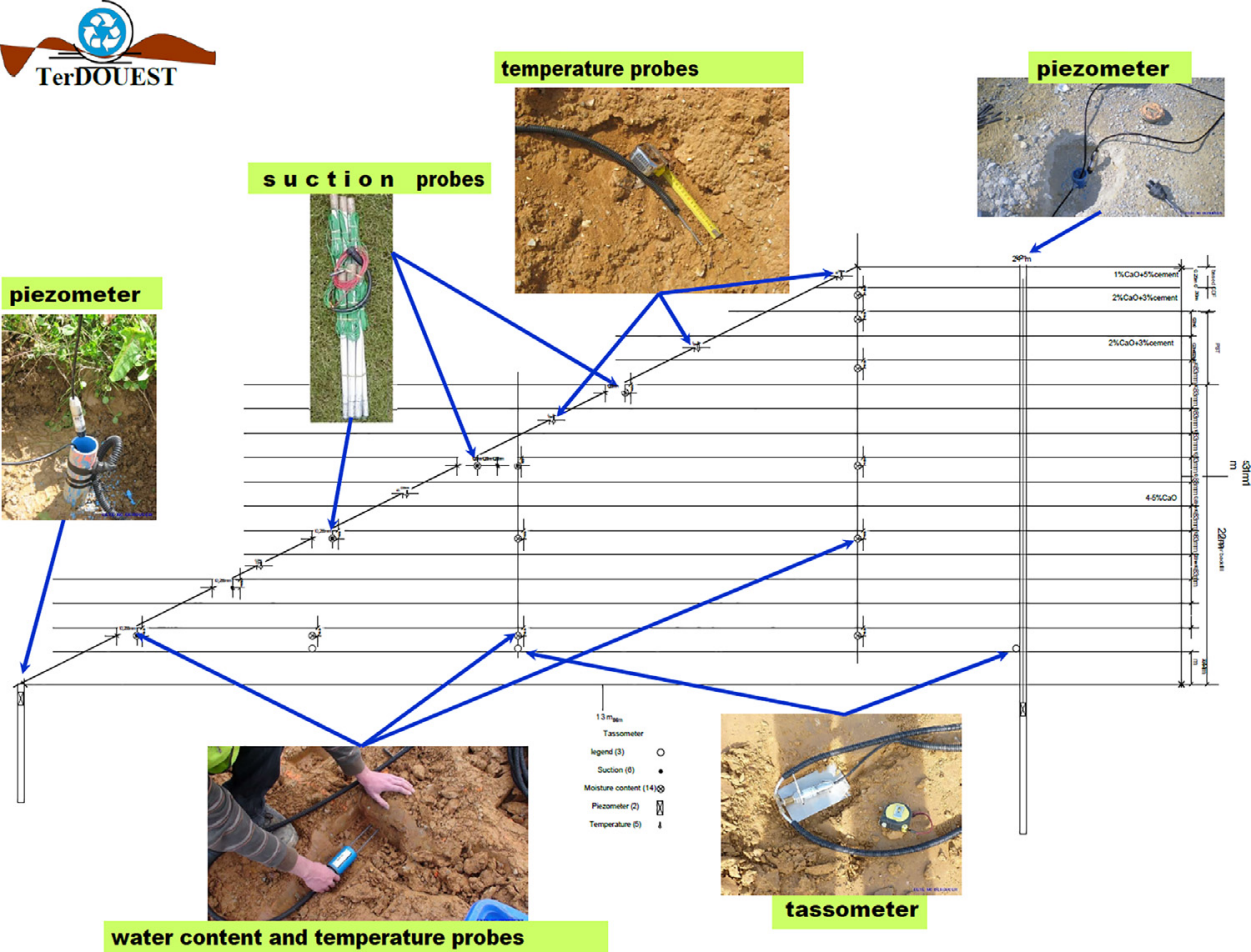
Probes location cross-section with some illustration.

## 9 The Suctions Probes

**Fig. 10:**
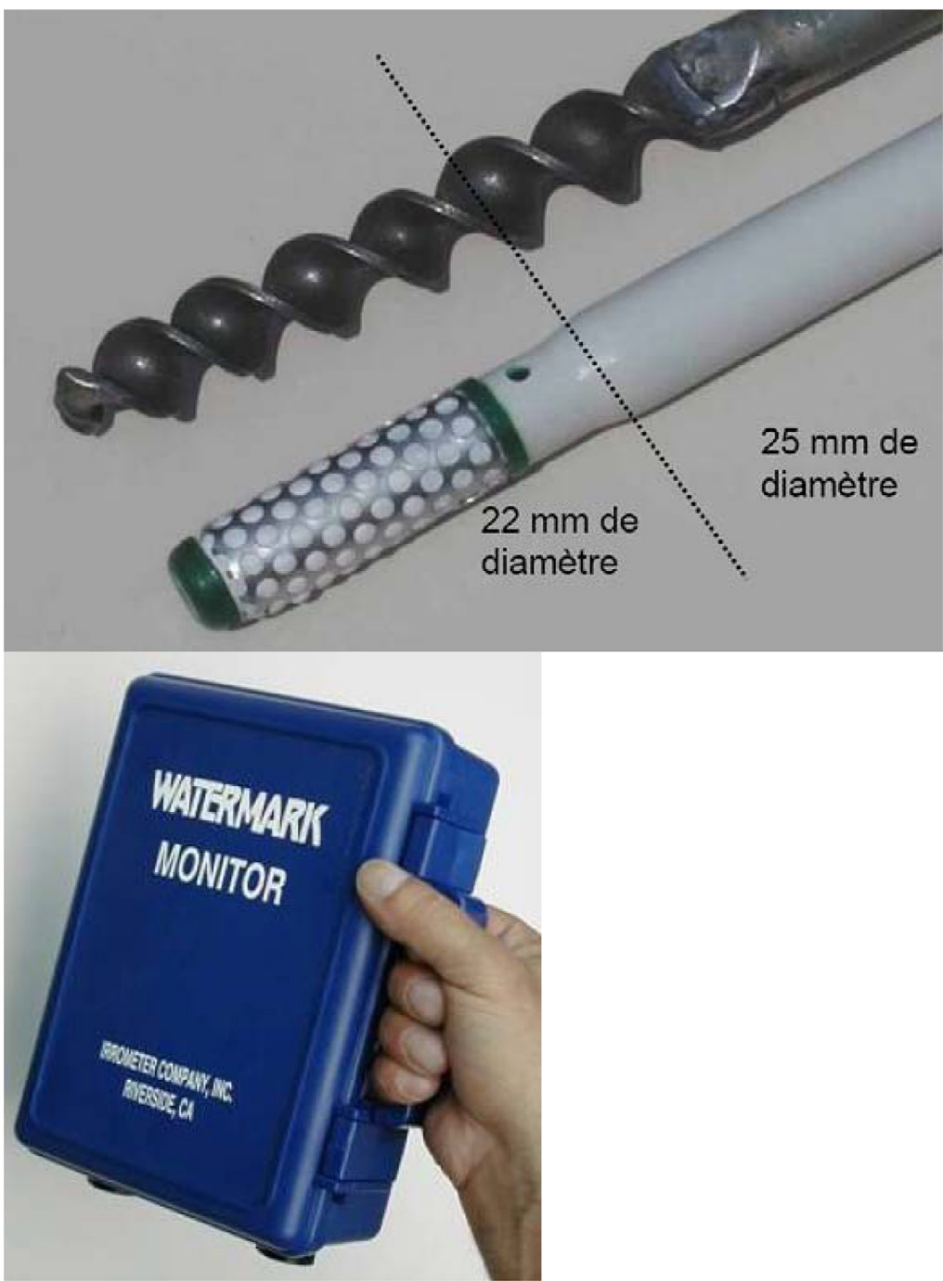
(a) 200 SS tensiometer probe, made by Watermark and (b) the installation kit (© Challenge Agriculture).

The measurements were taken using 200 SS tensiometer probes made by Watermark and distributed in France by Challenge Agriculture. The probes are coupled in batches of 6 to a Monitor-type datalogger. The technical specifications of the pressure used are as follows (source: Challenge Agriculture data) and illustrations are given in Fig. 10:

• Measuring range: 0-200 kPa;
• probe diameter: 22 mm;
• probe length: 83 mm;
• probe weight: 67 g
• accuracy of measurements: the constructor gives an in situ coefficient of variation of 5% for the same batch of probes. Constructor estimates that a value should be the mean value of at least 3 pairs of probes (total of 6 probes) which is not the case here.
• probe longevity: the manufacturer estimates a normal service life of around 7 years in use. In this experience, probes are still acquiring data after 13 years.
• Drift over time: there is no information from the manufacturer about a potential drift over this time but it may be a consequence of a long-term use as it is the case in this experience. In Fig. 3, Fig. 4 we can observe that low values of suction in the saturated domain slowly increased over time and never returned close to zero.

The probes were installed after the embankment had been built and the slope downstream of the embankment re-cut and after the 0.20 m layer of topsoil had been laid to cover the entire slope. The topsoil is removed manually where the collectors are to be then put back in place.

**Fig. 11:**
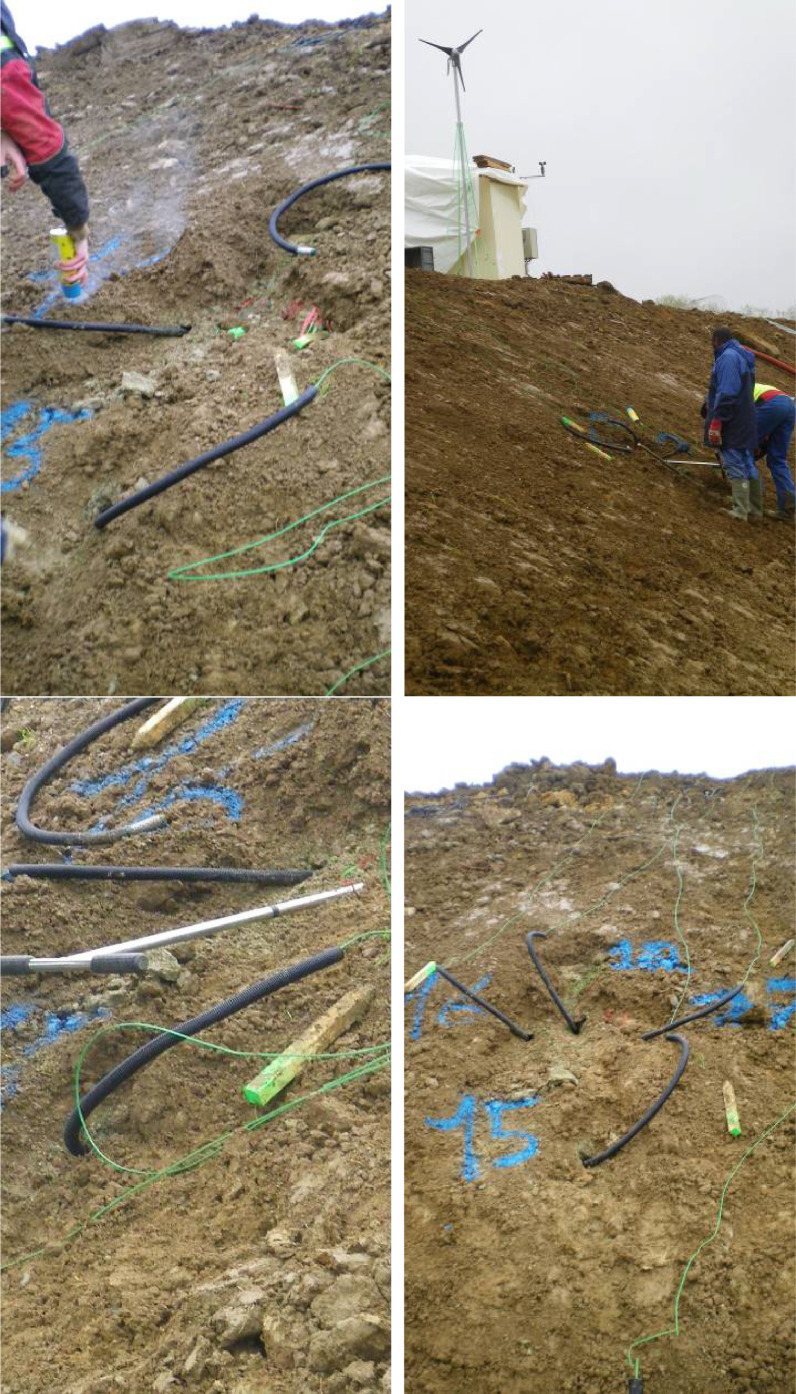
(a) Location of succion probes (b) general overview of the slope, in front of R8 layer (c) drilling of the preliminary hole before digging the probes (d) last view before overtopping with topsoil.

A preliminary hole is drilled with a hand auger (25 mm diameter), then the probes are manually sunk to the required depth. The photographs in Fig. 11 show the installation of the suction sensors on the slope of the experimental structure.

The data collected by each datalogger is sent by e-mail (approximately every two days) to the recipient.

## 10 The Piezometric Data

**Fig. 12:**
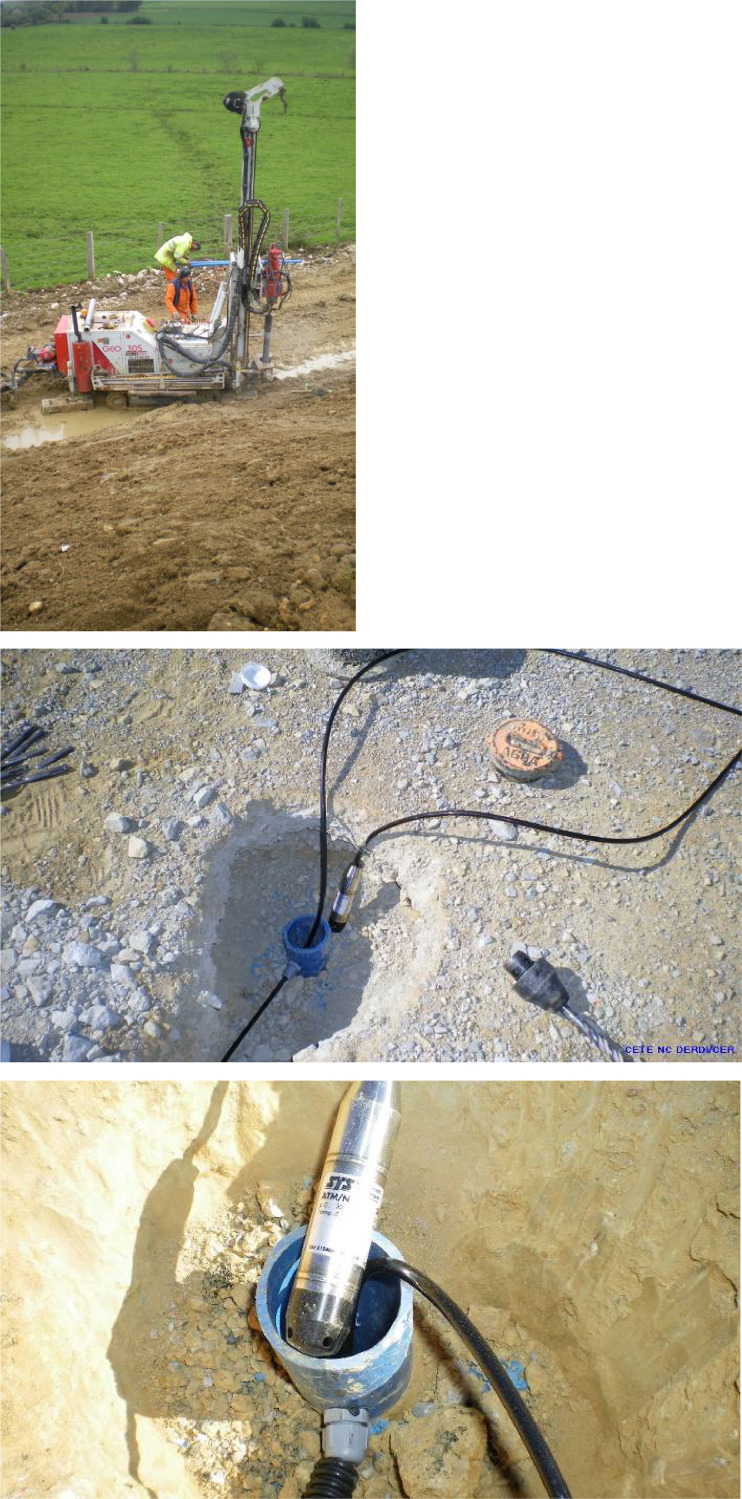
(a) Drilling a hole at the toe of the embankment, for bottom ground water measurements (b) clearing a drilling head to install the sensor in the tube (c) installing the pressure sensor in the tube.

The depth of the water table is measured using pressure sensors installed in piezo tubes drilled into the embankment (at the crest / the head of the embankment and at the toe / the bottom of the embankment). These sensors measure the pressure (and therefore the height) of the water above the level at which the sensor is immersed. By recording the various measurements when the sensors are installed (depth of the sensor in relation to the tube head and height of water above the sensor), the NGE level of the water table can be monitored over time. The photographs in Fig. 12 show the installation of the sensors used to measure the height of the water table.

**Fig. 13:**
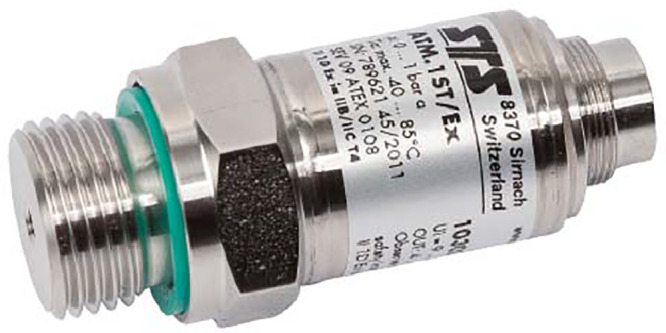
STS ATM pressure transmitter used for piezometric measurements.

The technical specifications of the pressure transmitter used are as follows and illustrations are given in Fig. 13:

• Measuring range: 0-100 mbar;
• Error : ≤ 0.25 % from the measuring range
• Temperature specification: -5 / +50°C;

## Limitations

Not applicable.

## Data Availability

Soil suction dataset from a lime & cement treated embankment, from 2010 to 2023 (Original data)
https://entrepot.recherche.data.gouv.fr/
